# Structural and Mutagenic Analysis of the RM Controller Protein C.Esp1396I

**DOI:** 10.1371/journal.pone.0098365

**Published:** 2014-06-02

**Authors:** Richard N. A. Martin, John E. McGeehan, Geoff Kneale

**Affiliations:** Biophysics Laboratories, School of Biological Sciences, Institute of Biomedical and Biomolecular Science, University of Portsmouth, Portsmouth, United Kingdom; MRC National Institute for Medical Research, United Kingdom

## Abstract

Bacterial restriction-modification (RM) systems are comprised of two complementary enzymatic activities that prevent the establishment of foreign DNA in a bacterial cell: DNA methylation and DNA restriction. These two activities are tightly regulated to prevent over-methylation or auto-restriction. Many Type II RM systems employ a controller (C) protein as a transcriptional regulator for the endonuclease gene (and in some cases, the methyltransferase gene also). All high-resolution structures of C-protein/DNA-protein complexes solved to date relate to C.Esp1396I, from which the interactions of specific amino acid residues with DNA bases and/or the phosphate backbone could be observed. Here we present both structural and DNA binding data for a series of mutations to the key DNA binding residues of C.Esp1396I. Our results indicate that mutations to the backbone binding residues (Y37, S52) had a lesser affect on DNA binding affinity than mutations to those residues that bind directly to the bases (T36, R46), and the contributions of each side chain to the binding energies are compared. High-resolution X-ray crystal structures of the mutant and native proteins showed that the fold of the proteins was unaffected by the mutations, but also revealed variation in the flexible loop conformations associated with DNA sequence recognition. Since the tyrosine residue Y37 contributes to DNA bending in the native complex, we have solved the structure of the Y37F mutant protein/DNA complex by X-ray crystallography to allow us to directly compare the structure of the DNA in the mutant and native complexes.

## Introduction

Restriction-modification (RM) systems encode a restriction endonuclease (ENase) and a DNA methyltransferase (MTase). The DNA MTase protects the host DNA from cleavage by the associated restriction enzyme, whilst the ENase cleaves foreign DNA that attempts to enter the bacterial cell, before it has time to be protected by methylation [1], [2]. Control mechanisms exist to ensure the correct temporal expression of RM genes, so that all recognition sites on the host DNA are methylated prior to exposure to the ENase.

The most widespread of these mechanisms employs a “controller” (C) protein encoded by a gene downstream of its own promoter, and usually co-transcribed with the endonuclease (R) gene as a single transcriptional unit [3]–[7]. The C-protein binds at various sites within the C/R promoter to regulate transcription of its own gene and the associated endonuclease gene [8]. ENase expression has been shown to be delayed with respect to the MTase when the C-protein is expressed in a new host *in vivo* [9]. Transcription of the C-gene is itself dependent on the concentration of the protein it encodes, leading to a regulatory feedback circuit [10].

**Figure 1:**
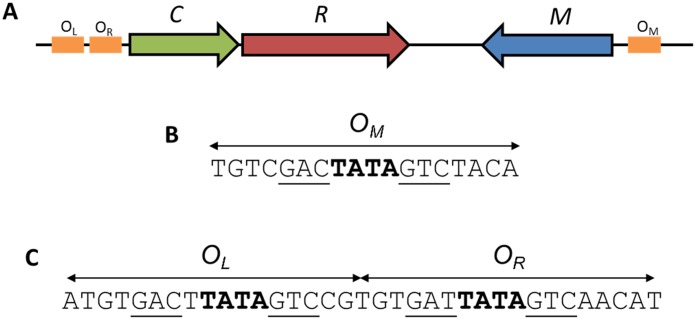
Gene arrangement of the Esp1396I RM system and C.Esp1396I binding sites. (A): The C-protein (C), endonuclease (R) and methyltransferase (M) genes. C/R genes form an operon that is convergent with the M gene. The three C-protein binding sites are shown in orange. (B) The O_M_ operator controls expression of the M gene. (C) The O_L_ and O_R_ operators control the expression of the C/R genes.

In the C.Esp1396I system, and other related systems, the operator sequence at the C/R promoter has two operator sites (denoted O_L_ and O_R_) that are distal and proximal, respectively, to the transcription unit comprised of the C and R genes [11], [12]. The high-affinity O_L_ site binds a C-protein dimer and recruits the sigma subunit of RNA polymerase to switch both the C and R genes on. As the C-protein concentration rapidly increases, the low affinity O_R_ site proximal to the gene then becomes occupied and the gene is down-regulated through displacement of bound RNA polymerase [12]–[14]. Unusually, in the RM system Esp1396I (Figure 1), the C-protein also represses the MTase (M) gene by binding to the promoter at the transcriptional start site, denoted O_M_ where the C/R genes and the M gene are transcribed convergently from different promoters [15].

Analysis of C-protein binding sites in a wide variety of RM systems suggested a repeating quasi-symmetrical “consensus” sequence consisting of two sets of inverted repeats or “C-boxes” upstream of the C/R genes [6], [8], [12], [16]. The first published structure of a controller protein bound to DNA was that of C.Esp1396I bound as a tetramer, i.e. with two dimers bound adjacently on the 35 bp operator sequence (O_L_+O_R_) of the C/R promoter to form the “repression complex” [11]. The structure revealed the mechanism of the switch from activation to repression of the C and R genes. In the crystal structure of the complex (PDB code: 3CLC), two dimers are bound to the DNA, each centred on the pseudo-dyad located between the central A and T bases in the TATA sequence within each operator site, and interacting across the major groove at the centre of the DNA.

Subsequent high resolution crystallographic studies of the complex with the O_L_ and O_M_ operators [17], [18] showed the sequence-specific contacts to the bases within the recognition site (“direct readout”), as well as the non-specific interactions with the severely bent phosphodiester backbone (“indirect readout”). We also investigated the affinities of the protein for its three natural promoters, O_M_, O_R_ and O_L_, in order to understand the structural and mechanistic basis of differential DNA sequence recognition in this system [18].

Here, we are interested in dissecting the contribution to DNA binding of key amino acid side chains involved in sequence specific interactions with the bases (T36, R46), and those involved in interactions with the DNA backbone (Y37, S52). By individually mutating these residues, and assessing DNA binding affinity by SPR, we analyse the contribution of the mutated side chains to DNA binding. To confirm that these mutations have not affected the overall fold of the protein, we have determined their structures by X-ray crystallography, and these structures show further conformational details of the flexible loop region involved in DNA sequence recognition. We also crystallized one of the mutant proteins bound to DNA and have determined the structure of this DNA-protein complex, to compare the conformation of the DNA in the native and mutant complexes.

## Results

### SPR Analysis of DNA Binding

**Figure 2:**
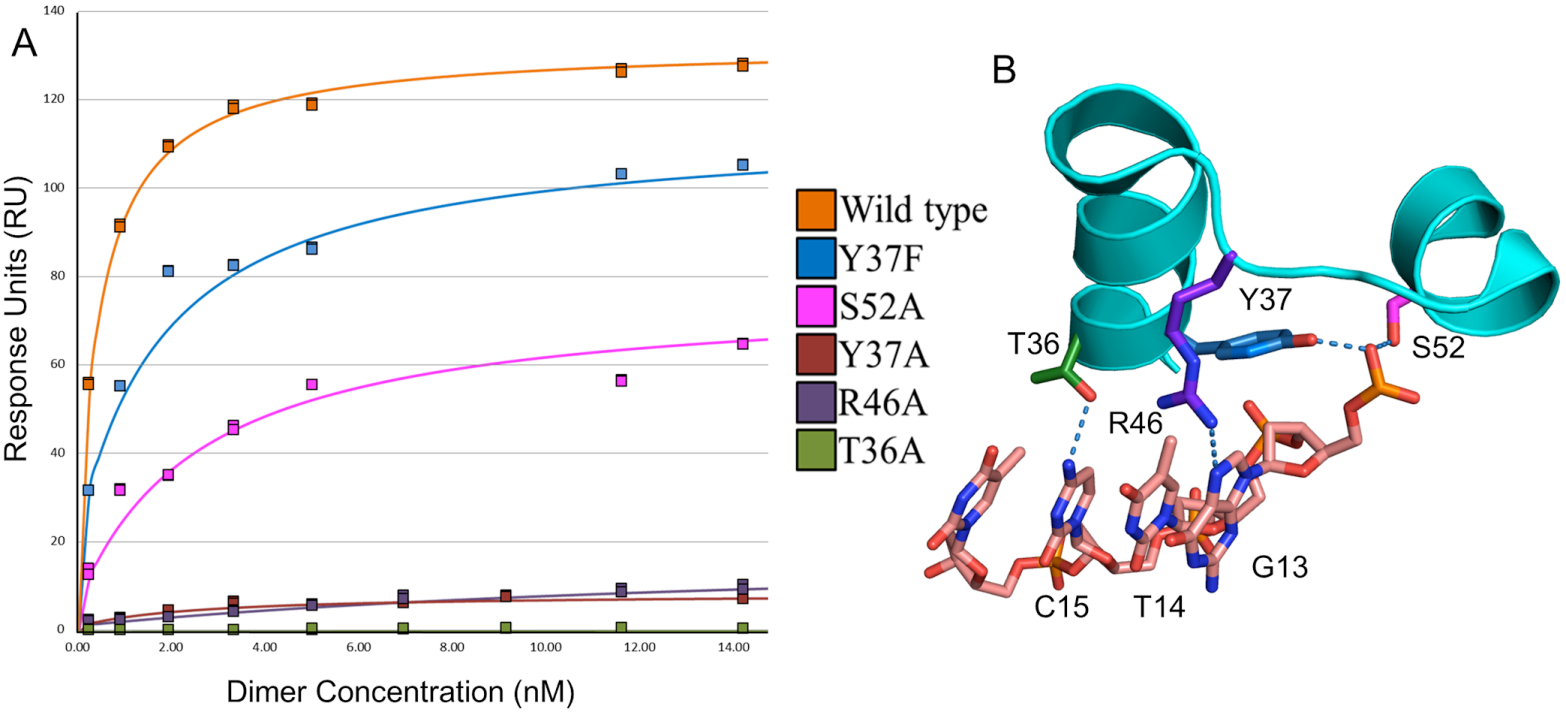
Binding analysis and location of mutations. (A) DNA binding curves of C.Esp1396I mutant proteins binding to the O_M_ operator site from SPR data. (B) The amino acid residues mutated in this study (T36, Y37, R46 and S52) showing their location at the DNA-protein interface. Image generated in PyMOL using the wild type C.Esp1396I-19O_M_ co-crystal structure (3UFD).

To evaluate the effects of the individual mutations on binding DNA, we used Surface Plasmon resonance (SPR) to follow binding to the O_M_ operator DNA sequence (see Figure S1). Since it was not possible in all cases to obtain accurate on- and off- rates, we determined the binding affinity of the mutants (and wild type) proteins from the SPR signal (RU) at equilibrium, using a range of protein concentrations (Figure 2A). The location of the mutated residues with respect to the bound DNA is shown in Figure 2B. The range of protein concentrations used for the SPR experiments varied from 20–200 nM total protein, corresponding to 0.2 nM to 17 nM dimer (see Methods section) and all except T36A were shown to interact with the O_M_ operator site within this concentration range. In further experiments, the maximum total protein concentration was increased to 1000 nM (corresponding to 210 nM dimer) but still no interactions were observed with those mutants (data not shown). The wild type protein, as expected, showed the highest binding affinity (*K_D_* = 0.5 nM). Of the mutant proteins, Y37F showed the strongest binding to the O_M_ site, followed by S52A and Y37A. The mutant protein R46A bound with a much lower affinity (∼16 nM), and T36A did not show any measurable binding under these conditions (Figure 2 and Table 1).

**Table 1:** Equilibrium SPR analysis of the C.Esp1396I mutant proteins binding to the O_M_ operator.

Protein	*K_D_* (nM)	Error (nM)	Chi^2^	ΔG (kJ mol^−1^)
**Wild type**	0.51	±0.04	1.90	−53.0
**Y37F**	1.80	±0.26	7.20	−49.9
**S52A**	2.60	±0.45	4.80	−49.0
**Y37A**	5.50	±1.70	0.16	−47.1
**R46A**	15.8	±4.70	0.18	−44.5
**T36A**	N/D	N/D	N/D	N/D

Analysis of the SPR data was performed using the 1∶1 affinity model from the BiaEval software. The dimerisation constant of C.Esp1396I was fixed at 1.6 µM to enable estimation of the dimer concentration from the total protein concentration. N/D = not detectable.

The *K_D_* for the R46A mutant was ∼30 fold greater than that of the wild type, showing a much weaker interaction with the O_M_ operator site, and consistent with a key DNA-binding role for this arginine side chain. An even more important role is indicated for T36, since the DNA binding ability of the T36A mutant was completely abolished, as measured by SPR. The *K_D_* for the S52A interaction with the O_M_ operator was 5 fold higher than the wild type, confirming the importance of this hydrogen bond interaction in stabilising the DNA-protein complex. The Y37A and Y37F mutants had *K_D_*s that were 11 and 3.5 fold higher than the wild type respectively. The three-fold difference between these two mutants indicates that both the phenyl ring and the hydroxyl group of the tyrosine are important for interacting with the DNA.

### Structural Analysis of Native and Mutant Proteins

In order to confirm that the structures of the mutant proteins closely resembled the native protein, and that the amino acid substitution had not introduced any inadvertent structural changes, we crystallized each mutant protein and solved their structures by molecular replacement. Since the resolution of the diffraction data from each mutant protein (1.5–2.0 Å) was significantly better than the previously determined native structure (2.7 Å), we also crystallized the native protein and obtained crystals that diffracted to much higher resolution (1.4 Å) than the published structure, to permit a more accurate comparison.

Overall the new triclinic wild type and mutant protein structures closely resemble the original protein structure [19], [20]. The root mean square deviations (RMSD) between the main chain atoms of each of the structures presented here and the previous structure (3G5G) were <1 Å in all cases. One obvious feature of the higher resolution structures was the presence of several water molecules on the surface of the proteins. The side chain positions could also be placed with a higher degree of confidence. Because the mutants crystallized in multiple space groups and unit cells, the crystal contacts were mostly unique to each structure. One key crystallisation contact in the majority of the structures relied upon an SO_4_ ion contacting symmetry related chains. The B-factors across the mutant structures are all fairly consistent with the C-terminus having the highest B-factors.

#### The native protein

**Figure 3:**
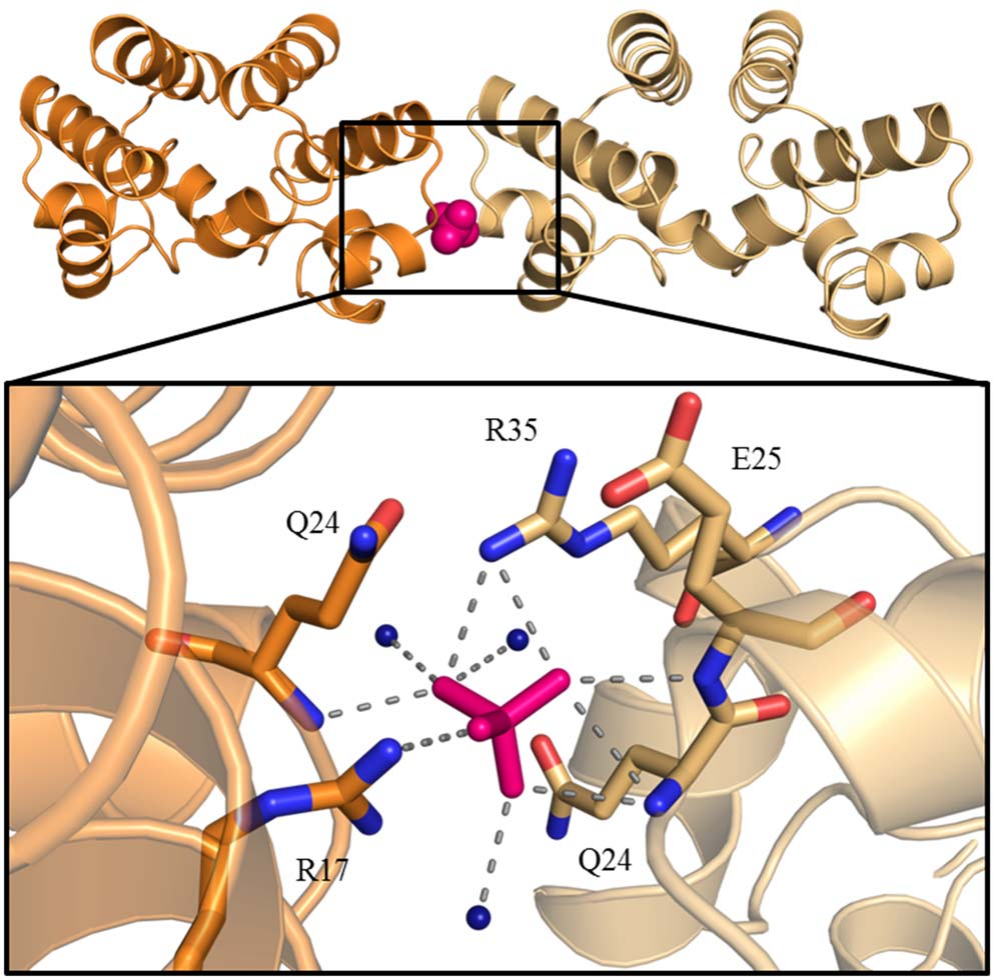
Coordination of the sulphate ion required in the crystal structure. Top panel shows the asymmetric unit of the C.Esp1396I high resolution wild type X-ray crystal structure (4I6R) in orange with a symmetry related dimer in light orange. The sulphate ion is shown in pink. The zoom shows atoms hydrogen bonding directly to the sulphate including water molecules (dark blue spheres). Hydrogen bonds are shown as grey dashed lines.

The high-resolution (1.4 Å) triclinic wild type structure presented here differs significantly in crystal contacts when compared to the previously described wild type structure 3G5G [19]. In terms of crystal contacts, Y29 does not stack with a symmetry related Y29 residue in the manner observed in several C.Esp1396I crystal structures. Instead the majority of the contacts are direct hydrogen bonds between side chains. A large number of crystal contacts occur through the C-terminal tail of C.Esp1396I and symmetry related chains. This gives that previously flexible region a high degree of stability, allowing the complete C-terminus up to the terminal carboxyl oxygen atoms to be modelled. The other primary intermolecular interface is centred on one of the SO_4_ ions. The SO_4_ ion essentially bridges the gap between symmetry related chains as in this region no direct protein-protein interactions occur (Figure 3). Of these intermolecular contacts, only R35 of the recognition helix is involved in DNA interactions in the protein-DNA complex. We see no difference in protein structure in different crystal forms of mutant and native proteins, regardless of the presence or absence of an SO_4_ ion in the unit cell.

**Figure 4:**
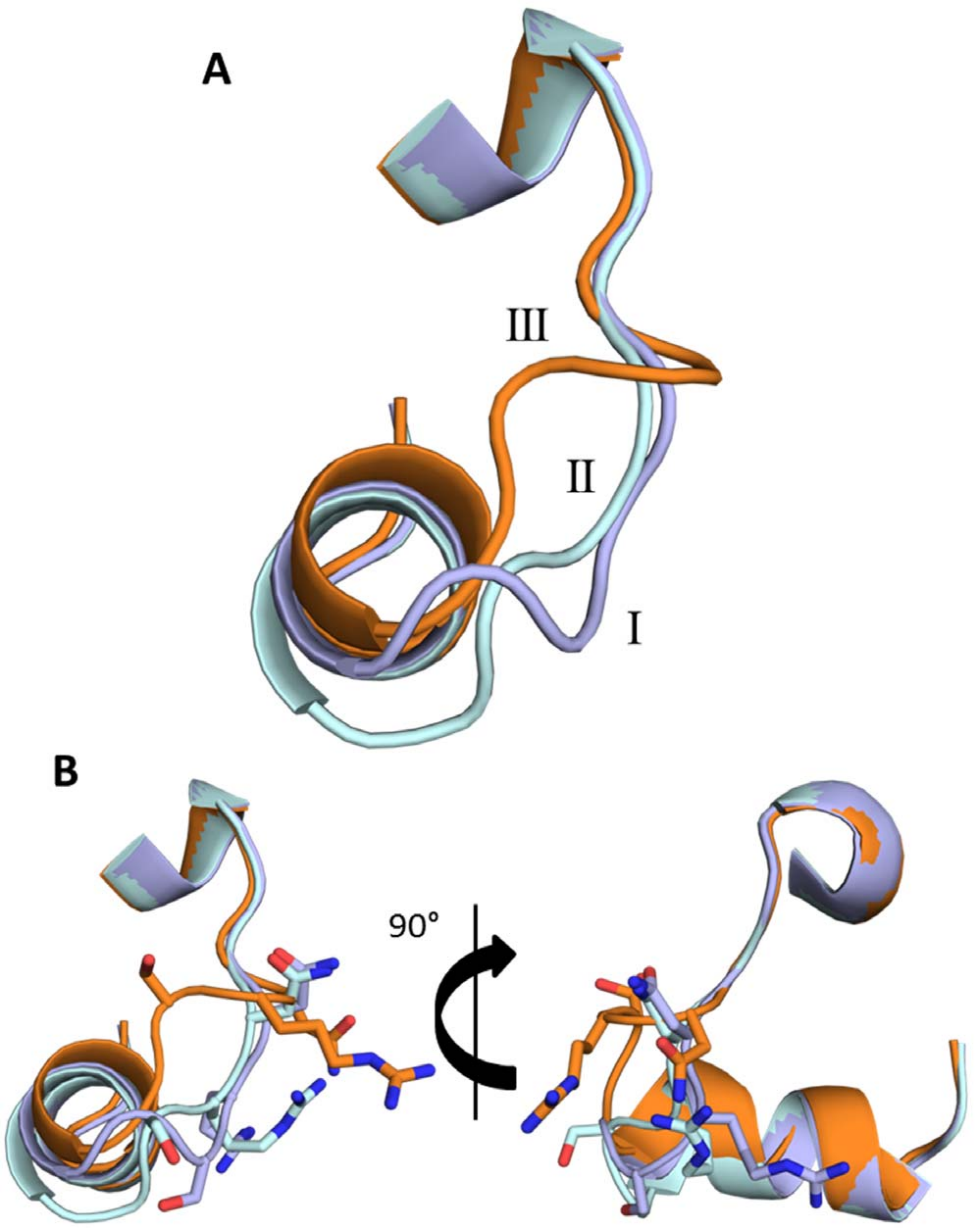
Conformation of the flexible loop of C.Esp1396I. (A): The three observed loop conformations observed in crystal structures of the native protein. The two alternative C.Esp1396I loop positions in 3G5G are shown in light blue (conformation I) and light green (conformation II) and the high resolution wild type loop position from 4I6R is shown in orange (III). (B): The side chains of residues S45, R46 and N47 are displayed as sticks.

**Figure 5:**
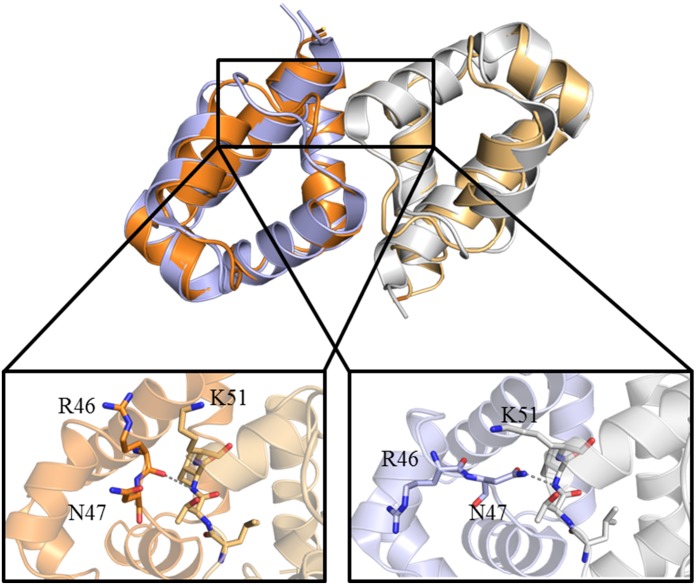
Dimerisation contacts in the alternative loop conformations. The high resolution wild type crystal structure (4I6R) is shown in orange (one monomer dark and one light) and the low resolution wild type is shown in blue/silver (one monomer dark and one light). The hydrogen bond is shown as a grey dashed line.

The flexible loop region identified by Ball *et al.* [19] is found in a different conformation to those previously described for this protein. Instead, the loop adopts another geometrically favourable position involving an ∼8 Å shift in the C_α_ of S45 (Figure 4). For clarity, the three loop positions of the protein will be referred to as I and II (the major and minor conformations from Ball *et al.* [19]) and III (the newly described loop conformation). The movement of the C_α_ of residue R46 between loop conformations I and III is ∼6 Å, resulting in an even larger movement of the R46 side chain (Figure 5). Since R46 is a key residue mediating DNA sequence recognition, this movement allows the recognition of variations on the consensus sequence of the C-box [18], [19].

#### Mutant protein structures

The mutant proteins Y37F, Y37A, T36A, S52A and R46A were crystallized (the latter in two different space groups) and the crystals diffracted X-rays to a resolution of 1.5–2.0 Å (Table 2). The SO_4_ based interaction observed in the high-resolution wild type structure is also observed in the Y37A, S52A and monoclinic R46A structures. Both the monoclinic R46A and S52A structures show almost identical crystal contacts to those of the triclinic wild type protein, even though the space group and unit cell are different. The other three mutant structures are all unique in space group, unit cell and crystal contacts. Despite Y37F and T36A only crystallising in the presence of SO_4_, this anion was not visible in either electron density map. As with the high-resolution wild type structure, the C-terminal tail is also involved in several crystal contacts with multiple monomers in the T36A, Y37F and trigonal R46A structures.

**Table 2:** Data collection and statistics from the C.Esp1396I and mutant free protein crystals.

Protein	Wild Type	T36A	Y37A	Y37F	R46A	R46A	S52A	Y37A-19O_M_
**PDB code**	4I6R	4I6T	4IA8	4I6U	4F8D	4FBI	4FN3	4IVZ
**Space Group**	*P1*	*P6_5_*	*P1*	*P2_1_2_1_2_1_*	*C2*	*P3_2_*	*C2*	*P2_1_*
	**Cell Dimensions:**							
**a, b, c (Å)**	34.76 34.78 41.86	65.45 65.45 72.29	34.26 34.28 41.12	48.61 81.85 135.08	46.40 51.00 74.60	65.33 65.33 71.38	46.59 51.32 74.38	47.50 146.70 47.80
**α, β, γ (°)**	110.47 105.60 96.00	90 90 120	104.44 108.92 97.42	90 90 90	90 96.60 90	90 90 120	90 95.46 90	90 93.21 90
**Solvent content (%)**	37.2	40.1	37.2	37.5	36.1	36.3	36.5	45.0
**Monomers in ASU**	2	2	2	6	2	4	2	4 (+4 DNA)
	**Data Collection:**							
**Beamline**	DLS-I04-1	DLS-I04-1	DLS-I04-1	DLS-I04-1	DLS-I02	DLS-I04-1	DLS-I02	DLS-I04-1
**Wavelength (Å)**	0.92	0.92	0.92	0.92	0.98	0.92	0.98	0.98
**Resolution (Å)**	1.4	2.0	1.9	2.0	1.5	1.5	1.8	3.1
**No. measured reflections**	112030	130244	44469	527601	81886	128533	96678	41888
**No. unique reflections**	29528	12474	11313	38881	27204	53279	30869	11810
**Completeness**	83.5 (83.8)	99.6 (100)	79.4 (82.6)	99.8 (100)	97.8 (94.9)	95.1 (97.0)	95.2 (94.3)	99.5 (99.6)
**<I/σ(I)>**	14.3 (4.0)	4.2 (1.1)	5.8 (1.5)	22.6 (6.7)	15.3 (3)	15.3 (3.9)	10.2 (3.2)	12.7 (1.8)
**Multiplicity**	3.8 (3.8)	10.4 (10.7)	3.9 (4)	13.6 (13.7)	3.2 (3.2)	2.4 (2.4)	3.1 (3.2)	3.8 (3.8)
***R_merge_*** **^†^**	4.3 (31)	41.8 (238.9)	11.0 (103)	8.0 (40.8)	4.0 (40.0)	3.4 (24.6)	6.5 (39)	9.8 (55.4)
CC_1/2_^*^	0.998 (0.881)	0.933 (0.549)	0.994 (0.705)	0.999 (0.963)	N/D	0.999 (0.881)	0.998 (0.895)	0.996 (0.701)
**Wilson B (Å^2^)**	10.64	18.92	21.54	16.87	23.01	14.61	19.34	58.46

Values in parenthesis are for the highest resolution shell. The CC_1/2_ value was not determined for the monoclinic R46A structure.

†
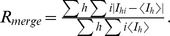

*
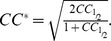

**Figure 6:**
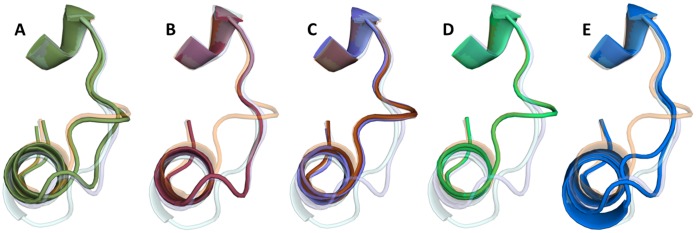
Conformation of the flexible loop of C.Esp1396I in the mutant crystal structures. The three wild type loop conformations observed in 3G5G and 4I6R are shown in the same colours as in Figure 5 and are translucent. (A) T36A loop positions shown in green. (B) Y37A loop position shown in dark red. (C) Loop positions in both R46A structures shown in red (monoclinic space group) and purple (trigonal space group). (D) Loop position of S52A shown in light green. (E) Loop positions in Y37F (blue).

The T36A mutant structure was observed to adopt two loop conformations (I and III) in equal proportions, indicating the conformational flexibility in this region. T36 is not part of the flexible loop region; it is in fact near the N-terminal end of helix 3 and therefore the likelihood of a mutation at this position altering the loop conformation is minimal. In terms of crystal packing interactions, the Y37A crystal structure is identical to the triclinic wild type structure but adopts loop conformation I in both monomers in the asymmetric unit. The Y37F crystal structure has six monomers (three biological dimers) in the asymmetric unit. Five of the monomers in the asymmetric unit are observed to be in loop conformation I but the sixth more closely resembles that of conformation II, which is almost identical to that in the DNA-protein complex with the 19O_M_ operator site [18]. Both of the R46A crystal structures and the S52A crystal structure have adopted loop conformation III, despite the varying buffer conditions and crystallographic interactions in those three structures. The ensemble of mutant loop conformations is shown in Figure 6.

#### Y37F-19OM Co-crystal structure

The DNA duplex was designed with overhanging A-T bases that, in the crystal, formed a pseudo-continuous DNA double helix. Intermolecular A-T basepairs were observed both within and between asymmetric units, identical to those observed in the WT-19O_M_ co-crystal structure. The protein-protein crystal contacts were also identical between the Y37F-19O_M_ and WT-19O_M_ structures. Very few dimer-dimer contacts were observed in the Y37F-19O_M_ crystal structure; however, one key contact occurs, involving Y29 of one chain stacking with Y29 of the symmetry-related chain, and additionally forms a stabilising hydrogen bond with D26.

**Figure 7:**
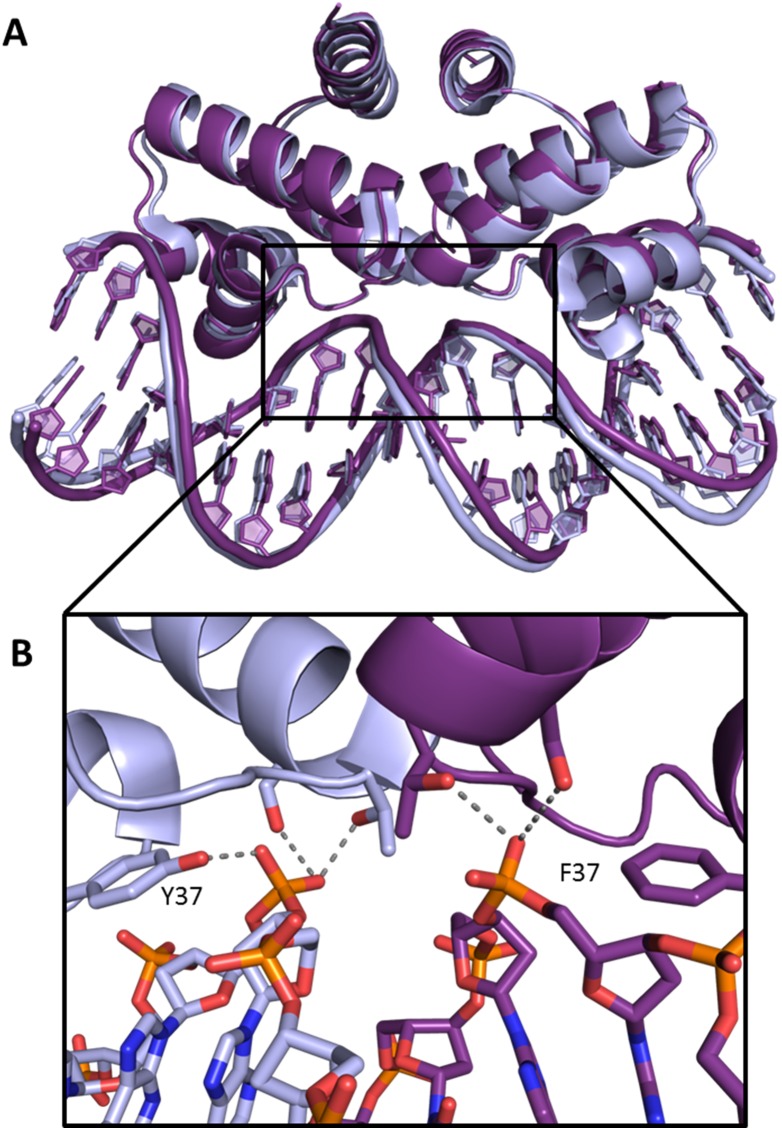
Comparison of the wild-type and Y37F mutant 19O_M_ co-crystal structures. The wild type structure is shown in pale blue and the Y37F mutant structure in purple. (A) An overlay of the two structures shows the high degree of similarity between the two. (B) The DNA “pinching” point at the central TATA sequence. Hydrogen bonds are shown as grey lines.

Comparing the two independent complexes in the asymmetric unit, the overall RMSD was 0.4 Å for all equivalent main chain atoms of the protein and the 18 DNA base pairs, and therefore only one complex is discussed further. The overall Y37F-19O_M_ structure was essentially identical to the native complex, with an RMSD of 0.5 Å for the main chain protein atoms. As with the native co-crystal structure, the flexible loop was identical in both protein subunits of the complex (Figure 7). The three major DNA base-binding residues R35, T36 and R46 were in essentially the same positions as those observed in the WT-19O_M_ co-crystal structure, making the same interactions.

**Figure 8:**
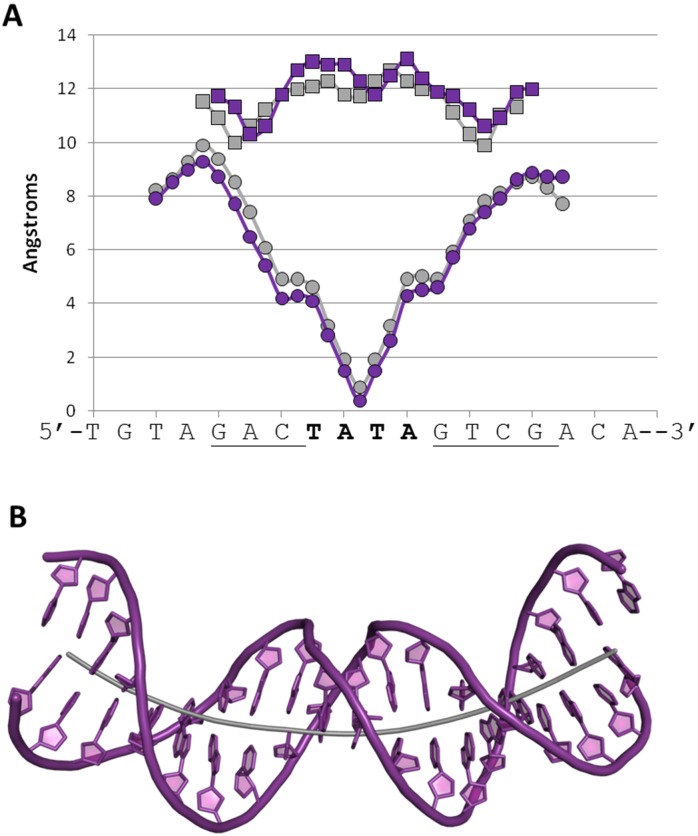
DNA bending analysis of the DNA from the C.Esp1396I-Y37F-19O_M_ co-crystal structure. (A) Groove width analysis of the DNA in the Y37F-19O_M_ crystal structure. The wild-type 19O_M_ co-crystal structure is shown in grey and the Y37F mutant co-crystal structure in purple. Major grooves widths are shown with squares and minor groove widths with circles. (B) The DNA duplex from the Y47F-19O_M_ co-crystal structure shown with the helical axis in grey. Analysis performed using the Curves+ server [32].

The structure of the DNA duplex in the Y37F-19O_M_ co-crystal structure also closely resembles that in the native complex, with an overall RMSD of 0.6 Å (Figure 8). The tyrosine residue Y37 in C.Esp1396I is implicated in DNA bending due to its interaction with the phosphate group at the highly compressed minor groove in all the known C-protein-DNA structures [11], [17], [18]. However, we find that the overall bend angles of the DNA in the Y37F-19O_M_ and WT-19O_M_ complexes are effectively identical at 54.7° and 56.0°, respectively [17]. This would suggest that the missing hydroxyl from the tyrosine, and concomitant loss of a key hydrogen bond interaction, does not affect the DNA distortion induced by C.Esp1396I.

## Discussion

SPR analysis showed that Y37F formed much more stable complexes with the O_M_ operator site than Y37A, with *K_D_*s of 1.8 and 5.5 nM respectively, but both were less stable than the wild type (*K_D_* = 0.51 nM). This indicated that the role of the tyrosine was more than just the formation of a hydrogen bond with the DNA backbone, but also involved the phenyl ring common to both tyrosine and phenylalanine. The free protein structures of the wild type, Y37F and Y37A mutants showed no major structural differences either in the local binding regions in terms of helix or side chain positions, or in the overall fold. Comparing the native and mutant (Y37F) protein-DNA complex structures, the tyrosine and the phenylalanine were equally buried and inaccessible to solvent and in both cases the phenyl ring stacks against the deoxyribose ring in the DNA.

From the Y37F-19O_M_ co-crystal structure, it is clear that the only major alteration to the binding interface is the loss of the hydroxyl - phosphate hydrogen bonds, of which there is one per protein subunit. The ΔG values were calculated from the equilibrium binding constants for the wild type, Y37F and Y37A proteins binding to DNA to be −53.0, −49.9 and −47.1 kJ mol^−1^ respectively (Table 1). The ΔG for the native C-protein-DNA of −53.0 kJ mol^−1^ falls within the range of other HTH DNA binding protein-DNA interactions including SinR (−36.7 kJ mol^−1^) [21], the C-protein C.AhdI (−45.7 kJ mol^−1^) (12) and the Cro repressor (−64.4 kJ mol^−1^) [22].

The ΔG difference (ΔΔG) due to loss of a side chain in the mutant proteins can be estimated. For the hydroxyl group only, the ΔΔG between the wild type and Y37F mutant was found to be −3.1 kJ mol^−1^ (−1.55 kJ mol^−1^ per hydrogen bond). The ΔΔG for the phenyl group alone (comparing the values for Y37F and Y37A), is −2.8 kJ mol^−1^ (−1.4 kJ mol^−1^ per monomer). Comparison of these two values indicates that the ring stacking and hydrogen bond interaction with the DNA make similar energetic contributions. Together, these interactions of Y37 with the DNA backbone contribute 5.9 kJ mol^−1^ per dimer. Comparing ΔGs for S52A and WT, we obtain ΔΔG of 4.0 kJ mol^−1^, comparable with that for the Y37F mutant (3.1 kJ mol^−1^), and indeed in the crystal structure of the protein-DNA complex, both of these residues form a hydrogen bond to the phosphodiester backbone of the DNA. The ΔΔG for R46A is significantly larger (8.5 kJ mol^−1^), and that for the T36A mutation must be larger still, since we could not measure any DNA binding activity of this mutant protein. Both R46 and T36 of C.Esp1396I are seen in crystal structures of DNA-protein complexes to interact directly with the base pairs involved in sequence recognition [18], specifically with the G and C bases, respectively, of the C-box recognition sequence GTC. Further hydrogen bonds from these amino acid residues to the thymine base may be mediated by water molecules.

The high-resolution structural studies of the mutants revealed the dimerisation interface of the proteins in great detail. Comparative analysis was then performed between these structures, previously published structures and the newly determined high-resolution (1.4 Å) wild type structure. It was observed that the dimerisation interfaces of the mutants were identical to the wild type protein, complete with the same hydrogen bonding networks and hydrophobic interactions.

As well as confirming that the mutant proteins were folded correctly, the structural studies revealed a more detailed structural picture of C.Esp1396I. Previously a flexible loop in C.Esp1396I had been identified from the wild type structures, where two alternative conformations were observed [19]. The wild type structure of C.Esp1396I presented in this study revealed further details including an additional, third conformation of the flexible loop region that was subsequently observed in several of the mutant protein structures. Between the 16 published and 20 newly determined structures of the C.Esp1396I monomer (from nine free protein X-ray crystal structures containing multiple monomers) and across seven different crystal forms, only these three loop conformations were observed. The T36A mutant protein crystal structure showed both the major conformation (I) and the novel conformation observed in this study (III), indicating that both could exist simultaneously in different subunits of the protein dimer. Flexibility in this loop is very important for DNA sequence recognition, as it has been observed in the nucleoprotein structures to adopt different conformations depending on which operator site the protein is bound to. Thus the side chains of DNA binding residues within the loop are presented in different orientations and so accommodate the different DNA sequences in the operator sites, albeit with very different affinities as required for finely tuned operation of the genetic switch control mechanism.

## Methods

Mutagenesis of C.Esp1396I to create the R46A and T36A mutant protein constructs was carried out as previously described [11]. All other mutant protein construct genes were synthesised by GenScript (USA). C.Esp1396I native and mutant constructs were purified as described previously [11]. In brief, all protein constructs were over-expressed in *E. coli* strain BL21 (DE3) pLysS with an N-terminal hexahistidine sequence for nickel affinity chromatography. After removal of the hexahistidine tag using the serine protease thrombin, size exclusion chromatography was used to further purify the C.Esp1396I proteins.

For SPR experiments the purified C.Esp1396I native and mutant proteins were dialysed into SPR running buffer (130 mM NaCl, 10 mM HEPES pH 7.4 and 0.05% (v/v) Tween-20). A 5′ biotinylated single stranded DNA oligonucleotide comprising the entire O_M_ operator site and its complementary sequence were synthesised by ATDBio (Southampton, UK). The two DNA molecules were incubated together at a 1∶1 molar ratio prior to heating to 353 K and cooling overnight. DNA duplexes were further purified using gel electrophoresis. The biotinylated DNA duplexes were diluted to 20 nM in the SPR running buffer prior to injection over a streptavidin coated SPR chip until a stable baseline of ∼200 response units (RU) was achieved. One channel was left empty to act as a control. Varying concentrations of the purified C.Esp1396I constructs (within the range 20–1000 nM total protein) were injected over the DNA coated chip and sensorgrams were recorded using a BIACore T-100. Data were processed using the BiaEval software with the total protein concentrations corrected to biological dimer concentrations using the dimerisation constant (*k_dim_*) of 1.6 µM previously reported [18].

Initial crystallisation experiments were carried out using the HoneyBee X8 crystallisation robot (Cronus Technologies) and sparse matrix screening using commercially available screens (Molecular Dimensions Ltd.). Proteins crystals were grown in the conditions summarised in Supplementary data (Table S1) at protein concentrations between 2 and 14 mg mL^−1^. For the Y37F-19O_M_ co-crystal structure, a DNA duplex corresponding to the published wild type 19O_M_ co-crystal structure [18] was synthesised as complementary single stranded DNA molecules, which were annealed and purified as for the SPR duplexes. The DNA duplex was incubated at varying molar ratios with the purified Y37F mutant C.Esp1396I construct prior to crystallisation trials. The addition of spermidine to a final concentration of 10 mM reduced precipitation in many conditions and resulted in larger crystals. Where required, crystals of sufficient quality for X-ray diffraction experiments were transferred to a cryo-protectant solution prior to flash-cooling in liquid nitrogen (Table S1). Cooled crystals were taken to the Diamond Light Source (Oxfordshire, UK) for X-ray diffraction experiments, all of which were conducted at 100 K. Details of data collection are summarised in Table 2. Diffraction data were processed using XDS and XSCALE [23] or MOSFLM [24] and Aimless [25], [26] and phases were obtained in all cases by molecular replacement with Phaser [27], [28] using the wild type monomer (3G5G) as a search model in each case (with the addition of the DNA duplex from 3UFD for the Y37F-19O_M_ structure). Refinement was carried out using iterative rounds of model building in Coot [29] and refinement in Refmac5 using TLS restraints [30]. Refinement statistics are summarised in Table 3. All refined structures were deposited into the Protein Data Bank with the accession codes detailed in Table2. Molecular images were produced using Pymol [31].

**Table 3:** Refinement statistics from the C.Esp1396I and mutant free protein crystals.*

Protein	Wild Type	T36A	Y37A	Y37F	R46A (*C2*)	R46A (*P3_2_*)	S52A	Y37F-19O_M_
**Refinement Parameters:**								
***R_work_*** **/** ***R_free_***	14.9/18.2	18.4/24.5	21.3/27.7	17.1/21.7	17.7/20.0	15.8/19.6	17.9/20.7	17.7/22.9
**No. atoms/B-factors (Å^2^)**								
**Protein**	1300/14.3	1312/28	1276/28	3797/23	1384/17	2750/15	1368/18	2482/71
**Water**	79/29	88/34	36/37	157/30	131/33	271/23	34/35	2/40
**Ligands**	45/28	7/57	5/24	37/39	22/38	12/23	15/32	N/A
**DNA**	N/A	N/A	N/A	N/A	N/A	N/A	N/A	1546/77
R.m.s. Deviations from standard*:								
**Bond lengths (Å)**	0.030	0.019	0.018	0.018	0.033	0.026	0.027	0.011
**Bond angles (°)**	2.70	1.88	1.83	1.86	2.63	2.88	2.17	1.716
**Ramachandran statistics (% of residues)** ¶								
**Favoured**	100	98.7	97.5	99.6	100	99.2	100	84.2
**Allowed**	100	100	100	99.8	100	100	100	99.7
**Outliers**	0	0	0	0.2	0	0	0	0.3

Standards as defined by Engh & Huber 1991 [33].

¶ As defined in MolProbity [34].

*ACCESSION NUMBERS: Coordinates and structure factors have been deposited in the Protein Data Bank with accession numbers 4I6R; 4I6T; 4IA8; 4I6U; 4F8D; 4FBI; 4FN3; 4IVZ.*

## Supporting Information

Figure S1 **Representative SPR data.** Sensorgrams for the wild type and mutant constructs of C.Esp1396I binding to the O_M_ operator site (200 nM total protein).
(PPTX)

Table S1 **Crystallisation conditions.**
(DOCX)
