# Supplementary material for: Structural and Mutagenic Analysis of the RM Controller Protein C.Esp1396I
Source: PLoS One. 2014 Jun 2;9(6):e98365. doi: 10.1371/journal.pone.0098365 (PMC4041747; doi:10.1371/journal.pone.0098365)
Supplement: Figure S1 — Representative SPR data. Sensorgrams for the wild type and mutant constructs of C.Esp1396I binding to the OM operator site (200 nM total protein). (PPTX) [file pone.0098365.s001.pptx]

## Slide 1
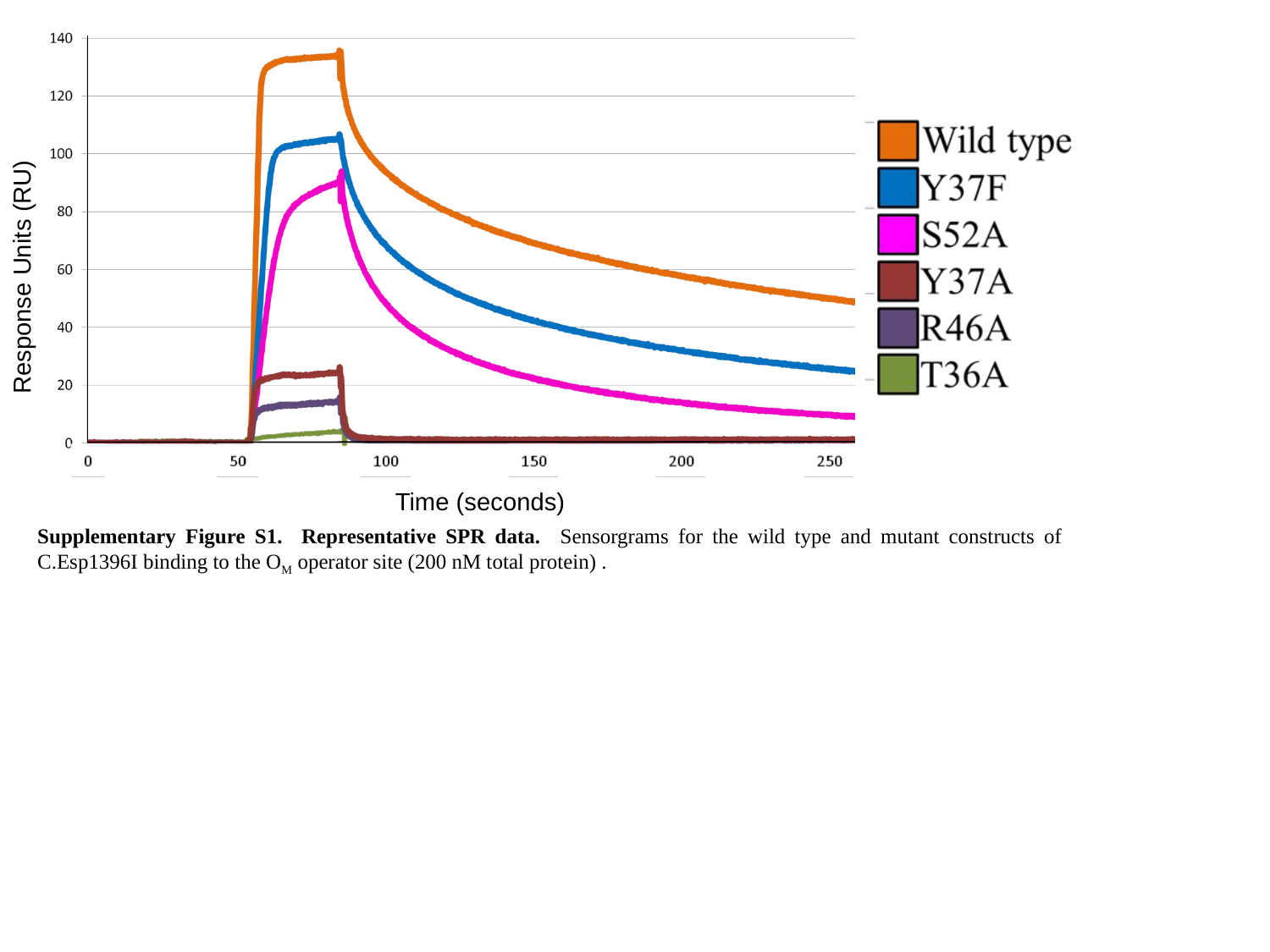

Time (seconds)
Response Units (RU)
Supplementary Figure S1. Representative SPR data. Sensorgrams for the wild type and mutant constructs of C.Esp1396I binding to the OM operator site (200 nM total protein) .
