# Supplementary material for: Structural and Mutagenic Analysis of the RM Controller Protein C.Esp1396I
Source: PLoS One. 2014 Jun 2;9(6):e98365. doi: 10.1371/journal.pone.0098365 (PMC4041747; doi:10.1371/journal.pone.0098365)
Supplement: Table S1 — Crystallisation conditions. (DOCX) [file pone.0098365.s002.docx]

| **Protein** | **Crystallisation condition** | **Cryo-protectant** |
| --- | --- | --- |
| C.Esp1396I wild type | 0.2 M lithium chloride, 0.1 M Tris-HCl pH 8, 20 % w/v PEG 6000 and 0.1 M sodium sulphate | 33 % v/v glycerol |
| C.Esp1396I T36A | 2.4 M sodium malonate pH 7 and 0.1 M sodium sulphate | 12.5 % di-ethylene glycol, 12.5 % glycerol and 12.5 % 1,2-propanediol |
| C.Esp1396I Y37A | 0.2 M lithium sulphate, 0.1 M sodium acetate pH 4.5 and 50 % v/v PEG 400 | N/A |
| C.Esp1396I Y37F | 0.2 M sodium acetate 0.1 M Bis Tris propane pH 8.5, 20 % w/v PEG 3350 and 0.1 M sodium sulphate | 12.5 % di-ethylene glycol, 12.5 % glycerol and 12.5 % 1,2-propanediol |
| C.Esp1396I R46A (monoclinic) | 0.2 M sodium sulphate, 20 % w/v PEG 3350 | 33 % v/v glycerol |
| C.Esp1396I R46A (trigonal) | 0.1 M MIB buffer pH 9 and 25 % w/v PEG 1500 | 33 % v/v glycerol |
| C.Esp1396I S52A | 0.2 M sodium sulphate, 20 % w/v PEG 3350 | 6.25 % di-ethylene glycol, 12.5 % ethylene glycol, 6.25 % MPD, 6.25 % 1,2-propanediol, 6.25 % glycerol and 6.25 mM NDSB 201 |
| C.Esp1396I Y37F-19O_M_ | 0.1 M SPG buffer (succinic acid, sodium dihydrogen phosphate, and glycine, 2:7:7) pH 8, 25 % (w/v) PEG 1500 and 10 mM spermidine | 33 % v/v glycerol |

**Supplementary Table S1. Crystallisation conditions**
